# Cohort profile: GRACE – a residential aged care cohort examining factors influencing antimicrobial resistance carriage

**DOI:** 10.1186/s12877-023-04215-3

**Published:** 2023-08-28

**Authors:** Lucy Carpenter, Andrew P. Shoubridge, Erin Flynn, Catherine Lang, Steven L. Taylor, Lito E. Papanicolas, Josephine Collins, David Gordon, David J. Lynn, Maria Crotty, Craig Whitehead, Lex E. X. Leong, Steve L. Wesselingh, Kerry Ivey, Maria C. Inacio, Geraint B. Rogers

**Affiliations:** 1 Microbiome and Host Health Programme, South Australian Health and Medical Research Institute, 5D332, Flinders Medical Centre, Flinders Drive, Bedford Park, Adelaide, SA 5042 Australia; 2 College of Medicine and Public Health, Flinders University, Adelaide, SA Australia; 3 National Centre for Epidemiology and Population Health, Australian National University, Canberra, ACT Australia; 4 Registry of Senior Australians, South Australian Health and Medical Research Institute, Adelaide, SA Australia; 5 SA Pathology, Adelaide, SA Australia; 6 Department of Microbiology and Infectious Diseases, Flinders Medical Centre, Adelaide, SA Australia; 7 Computational & Systems Biology Programme, South Australian Health and Medical Research Institute, Adelaide, SA Australia; 8 Southern Adelaide Local Health Network, SA Health, Adelaide, SA Australia; 9 Department of Nutrition, Harvard T.H. Chan School of Public Health, Boston, MA USA; 10 Allied Health and Human Performance, University of South Australia, Adelaide, SA Australia

**Keywords:** Infection control, Geriatric medicine, Microbiology

## Abstract

**Background:**

The emergence of antimicrobial-resistant bacteria represents a considerable threat to human health, particularly for vulnerable populations such as those living in residential aged care. However, antimicrobial resistance carriage and modes of transmission remain incompletely understood. The Generating evidence on antimicrobial Resistance in the Aged Care Environment (GRACE) study was established to determine principal risk factors of antimicrobial resistance carriage and transmission in residential aged care facilities (RACFs). This article describes the cohort characteristics, national representation, and planned analyses for this study.

**Methods:**

Between March 2019 and March 2020, 279 participants were recruited from five South Australian RACFs. The median age was 88.6 years, the median period in residence was 681 days, and 71.7% were female. A dementia diagnosis was recorded in 54.5% and more than two thirds had moderate to severe cognitive impairment (68.8%). 61% had received at least one course of antibiotics in the 12 months prior to enrolment.

**Results:**

To investigate the representation of the GRACE cohort to Australians in residential aged care, its characteristics were compared to a subset of the historical cohort of the Registry of Senior Australians (ROSA). This included 142,923 individuals who were permanent residents of RACFs on June 30th, 2017. GRACE and ROSA cohorts were similar in age, sex, and duration of residential care, prevalence of health conditions, and recorded dementia diagnoses. Differences were observed in care requirements and antibiotic exposure (both higher for GRACE participants). GRACE participants had fewer hospital visits compared to the ROSA cohort, and a smaller proportion were prescribed psycholeptic medications.

**Conclusions:**

We have assembled a cohort of aged care residents that is representative of the Australian aged care population, and which provides a basis for future analyses. Metagenomic data isolated from participants and built environments will be used to determine microbiome and resistome characteristics of an individual and the facility. Individual and facility risk exposures will be aligned with metagenomic data to identify principal determinants for antimicrobial resistance carriage. Ultimately, this analysis will inform measures aimed at reducing the emergence and spread of antimicrobial resistant pathogens in this high-risk population.

**Supplementary Information:**

The online version contains supplementary material available at 10.1186/s12877-023-04215-3.

## Background

In keeping with trends globally, Australia is experiencing significant ageing of its population [1]. By 2031, 21% of Australians will be aged over 65 years [2]. Of Australians over 65, 6% currently live in residential aged care facilities (RACFs), and of those 85 years and over, 30% do [2, 3]. The threat of increasing rates of infection caused by multidrug-resistant organisms (MDRO) is particularly serious in RACFs. High rates of antibiotic prescription, poor antimicrobial stewardship, and the potential for microbial transmission between residents, all contribute to growing rates of multidrug-resistant clinical isolates [4–6]. However, the prevalence of antimicrobial resistance (AMR) in asymptomatic individuals (carried either by pathogens or commensal microbes), or the dispersal of MDRO within the RACF environment, is largely uncharacterised. Despite serious concerns about a growing inability to readily treat common infections, and the potential for RACF populations to contribute to AMR carriage within the wider community, sufficiently detailed data to support the development of effective measures to limit the spread of MDRO in aged care simply do not exist.

The Generating evidence on Resistant bacteria in the Aged Care Environment (GRACE) study enrolled residents from five RACFs located in metropolitan Adelaide, South Australia. Our principal aim in establishing this cohort was to provide a basis for investigations of the distribution and determinants of antimicrobial resistance carriage in residential aged care. This will be addressed through future analyses that focus on five questions that are fundamental to developing effective strategies to reduce AMR carriage in RACF residents: (1) What exposures influence the types and levels of AMR determinants carried by RACF residents? (2) To what extent is there evidence of AMR transmission between RACF residents? (3) Is interaction with the RACF built environment likely to facilitate AMR transmission? (4) Do hospital visits for acute care significantly influence types and levels of AMR carriage? (5) To what extent do ageing-associated changes in gut microbiology influence AMR carriage?

To address these research questions, participants were invited to provide stool and oropharyngeal samples for metagenomic analysis to determine microbiome and resistome characteristics (glossary of terms listed in **Supplementary Table 1**), and comprise the cohort described in this article. Environmental samples were also collected from areas within each facility. Metagenomic data from this cohort will be related to a range of factors, including facility variables, resident demographics, morbidity, and polypharmacy data (**Supplementary Table 1**), to identify influences on AMR carriage and potential transmission.

Prior to these future analyses, we compared GRACE cohort characteristics with those of aged care residents within the national historical cohort of the Registry of Senior Australians (ROSA) which contains data for more than 2.8 million Australians aged over 65 who accessed government-subsidised aged services from 1997 to 2017 [7]. We present baseline data for the GRACE cohort, determine if the cohort was representative of the wider Australian aged care population, and its validity as a basis to provide further insight into AMR carriage nationally.

## Methods

### Study design and population

GRACE is a prospective, cohort study of permanent residents of RACFs recruited between March 2019 and March 2020. All eligible residents living in participating facilities at the time of recruitment and/or their next of kin were approached by a research nurse to provide informed consent. In addition to the consent form, study information was made available in the form of a video, a two-page brochure and on a website. Consent could be provided for one or all study procedures, including the collection of stool and/or oropharyngeal samples, collection of facility-level medical records and access to data held by the Medicare Benefits Schedule (MBS) and Pharmaceutical Benefits Scheme (PBS). Participants were not eligible if: (1) they were in respite care, (2) they were receiving palliative/end-of-life care, (3) it was recommended by management that they not be approached, and (4) we were unable to contact next of kin where third-party consent was required. Participants who required third-party consent, such as those with cognitive impairment, were identified by the participating facility and communicated to the study team. GRACE aimed to recruit 400 residents across 10 RACFs. However, due to the COVID-19 pandemic, and the imposition of strict facility entry restrictions, recruitment was ceased, resulting in a sample size of 279 residents from five facilities, with a mean recruitment rate of 75%. Site 1 was excluded from this mean as data on eligibility and participants who declined was not recorded.

**Fig. 1:**
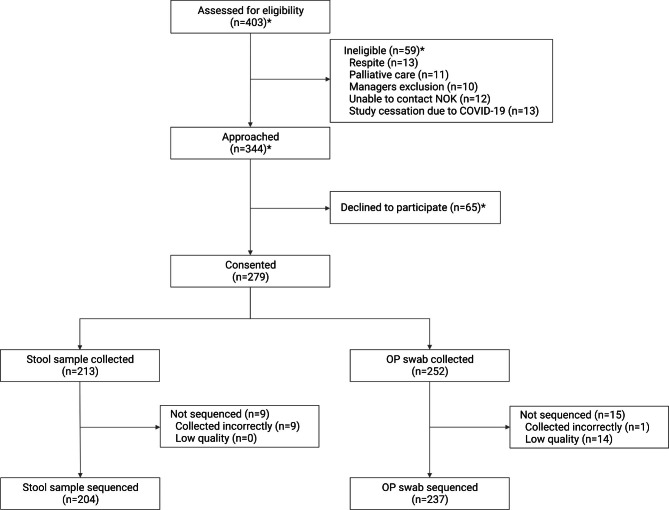
GRACE recruitment process. GRACE study recruitment and sample collection numbers. * data does not include site 1; NOK = next of kin; OP = oropharyngeal

Of 403 residents assessed for eligibility, 344 were approached to join the study and 279 consented to participate (Fig. 1). Fifty-nine residents were ineligible and 65 declined to participate (excluding site 1). Of those who consented, 111 (39.8%) provided consent themselves, and 168 (60.2%) provided third-party consent. Two-hundred and seventy-three residents (97.8%) provided consent for Department of Human Services (DHS) data access, with MBS and PBS data available for 243 and 228 residents, respectively.

### Data collection

Participant and facility data were collected at the close of recruitment at each site and included facility medical records. Information held by the PBS and MBS from the DHS was requested after all recruitment was complete. A summary of type and source of all clinical data collected is shown in Table 1. Demographical data (including age and sex), as well as data on participant living arrangements (time spent in current facility, room type, room security) were collected from facilities. In addition, data on care requirements was collected from facilities via the Aged Care Funding Instrument (ACFI), a tool used on entry to a RACF to determine the funding needed for a person’s care [8]. This includes three domains representing different areas of care needs: Activities of Daily Living, Cognition and Behaviour, and Complex Healthcare. Activities of Daily Living, includes details of care required for eating, showering, toileting and general mobility. Cognition and Behaviour domain measures the cognitive skills, verbal and physical behaviour, and mental health of individuals. Complex Healthcare considers the support residents need to manage their medications and health conditions. The ACFI also includes data on cognitive and behavioural conditions, which we have used to determine the presence of dementia in our cohort. Cognitive impairment scores pre-calculated using the Psychogeriatric Assessment Scales – Cognitive Impairment Scale (PAS-CIS) method were also obtained from the ACFI data [9]. Details of hospitalisations in the 12 months prior to enrolment, diet type and texture, and medical care data (wound care, medical devices) were collected from the facility records.

**Table 1:** Sources of clinical data collected for the GRACE Study

Category	Metrics	Source
**Demographics**	Age Sex Location Facility type	Facility records
**Built environment**	Time spent in current facility Room type Room security	Facility records
**Care requirements**	Activities of Daily Living Cognition and Behaviour Complex Healthcare Medical conditions Diet (type and texture)	Facility records (Aged Care Funding Instrument)
**Additional care**	Hospitalisations Wound care Medical devices	Facility records
**Medications**	Antibiotics Antidementia Antidepressants Antidiabetics Anti-inflammatories Antimycotics Antivirals Corticosteroids Hormones Immunosuppressants Insulin Lipid-modifying and beta-blocking agents Medicines for constipation and acid-related disorders Opioids Psycholeptics	Pharmaceutical Benefits Scheme (PBS)
**Healthcare usage**	Allied health service Diagnostic imaging General practitioner Health assessment Medical specialist Pathology Surgery	Medicare Benefits Schedule (MBS)

Data collected from the PBS included medications prescribed during the 12 months prior to study enrolment for each participant. Specifically, data were obtained relating to medications that might directly or indirectly influence the microbiome and care needs (antibiotics, antivirals, antimycotics, medicines for constipation and acid-related disorders, insulin, antidiabetics, opioids, anti-inflammatories, corticosteroids, immunosuppressants, hormones, lipid-modifying and beta-blocking agents, antidementia medication, antidepressants, and psycholeptics (includes antipsychotics, anxiolytics, sedatives/hypnotics)). Data collected from the MBS included healthcare services such as general practitioner (GP) attendances, specialist attendances, allied health services, surgery, diagnostic imaging services, health assessments, and access of pathology services during the 12 months prior to study enrolment for each participant (full definitions and coding of these variables in **Supplementary Table 2**).

RxRisk is an established tool to determine a person’s actively managed health conditions using their medication data and was used to compare health conditions between GRACE and ROSA [10]. In GRACE, RxRisk health conditions were able to be assessed for 228 participants as this relied on PBS data availability. Dementia is reported using both the RxRisk method and the ACFI diagnosis as per previously reported [7].

### Comparison with the national aged care data

Data from the National Historical Cohort of the Registry of Senior Australians (ROSA) was used to evaluate the extent to which the study cohort was representative of the national residential aged care population [7]. ROSA includes Australians aged 65 years and over who accessed government-subsidised aged care services between 1997 and 2017. ROSA has integrated information from the aged care sector with various health care data sources. Datasets within ROSA include: Australian Institute of Health and Welfare’s National Aged Care Data Clearinghouse datasets, Australian Government Medicare Benefits Schedule (MBS) and Pharmaceutical Benefits Scheme (PBS), state health authorities’ hospitalisations (QLD, NSW, VIC, SA), and ambulance datasets (NSW, SA). All data were de-identified and integrated by approved agencies (Australian Institute of Health and Welfare, Centre for Health Record Linkage, Centre for Victorian Data Linkage, SA NT DataLink and Queensland Health’s Statistical Services Branch). Details of ROSA datasets, variables, definitions, and limitations have been published previously [7]. The June 30th, 2017 (latest available data at the time of the study) non-Indigenous national cohort of permanent residents of RACFs (n = 142,923) was obtained from ROSA for comparison to the GRACE cohort. Analysis focusing on MBS subsidized health care services only included individuals without Department of Veterans’ Affairs cards (n = 123,555), and analysis focusing on hospitalization records only included individuals living in NSW, VIC, SA, and QLD (n = 125,351).

### Statistical analysis

Descriptive statistics were used to summarise characteristics of both GRACE and ROSA derived populations. For continuous data, median (IQR) was reported. For categorical data, percent and number of participants was reported. GRACE data was exported, cleaned, and analysed in Statistical Analysis Software (SAS) University Edition (SAS Studio v3.8/SAS v9.4).

## Results

### Demographics

A comparison of participant clinical data between GRACE (n = 279) and ROSA (n = 142,923) is shown in Table 2. GRACE participants had a median age of 88.6 (IQR = 81.8–93.2) years, which was similar to that of ROSA (med = 87.4, IQR = 81.6–91.7). GRACE and ROSA participants were mostly female (GRACE = 71.7%, n = 200; ROSA = 68.4%, n = 97,706), had a similar prevalence of dementia (GRACE = 54.5%, n = 152, ROSA = 53.6%, n = 76,594), and residents had been in their current facility for a similar period of time at recruitment/data collection (GRACE: med = 681 days, IQR = 252–1147; ROSA: med = 689 days, IQR = 283–1391). GRACE participants all lived in metropolitan facilities, run by not-for-profit organisations, whereas ROSA participants lived in a number of locations and organisation types. There are 155 urban facilities in total across South Australia that are either government, for-profit, or not-for-profit.

**Table 2:** Characteristics of GRACE study participants compared to the national population in ROSA

	GRACE (n = 279)	ROSA (n = 142,923)
**Demographics**		
Age, median (IQR) (years)	88.6 (81.8–93.2)	87.4 (81.6–91.7)
Sex, % (n)		
Female	71.7 (200)	68.4 (97,706)
Male	28.3 (79)	31.6 (45,217)
Facility location, % (n) †		
Major city	100 (279)	70.1 (100,140)
Outside major city	0 (0)	29.8 (42,525)
Organisation type, % (n) †		
Government	0 (0)	4.0 (5,787)
Not-for-profit	100 (279)	56.7 (80,992)
For-profit	0 (0)	39.2 (55,958)
Days lived in facility, median (IQR)	681 (252–1147)	689 (283–1391)
Dementia diagnosis, % (n)* †	54.5 (152)	53.6 (76,594)
**Care requirements (ACFI)**		
Activities of Daily Living, % (n)^ †		
High	65.9 (184)	54.3 (77,552)
Medium	26.5 (74)	30.9 (44,157)
Low	6.5 (18)	13.5 (19,280)
Nil	0 (0)	0.6 (874)
Cognition and behaviour, % (n)^ †		
High	47.0 (131)	60.3 (86,117)
Medium	33.0 (92)	22.8 (32,629)
Low	17.5 (49)	11.5 (16,502)
Nil	0 (0)	4.6 (6,615)
Complex healthcare, % (n)^ †		
High	64.5 (180)	53.3 (76,228)
Medium	28.3 (79)	28.6 (40,863)
Low	6.1 (17)	15.4 (22,066)
Nil	0 (0)	1.9 (2,706)
**Healthcare services, % accessed at least once in 12 months prior to enrolment (n)^**		
At least 1 healthcare service accessed, % (n)	100 (243)	99.4 (122,875)
GP attendance	38.7 (94)	45.7 (56,465)
GP attendance after hours	85.5 (207)	54.7 (67,643)
Specialist attendance	31.7 (77)	27.4 (33,822)
GP management plans, team care arrangements, multidisciplinary care plans	81.1 (197)	56.0 (69,213)
Collaborative domiciliary and residential management reviews	61.7 (150)	33.7 (41,696)
Diagnostic imaging (any, per resident)	51.4 (125)	44.6 (55,126)
Health assessments	64.6 (157)	44.8 (55,375)
Geriatric medicine	10.7 (26)	7.3 (9,038)
Urgent attendance after hours	61.7 (150)	33.1 (40,904)
Medical practitioner (emergency physician) attendance	3.7 (9)	2.0 (2,516)
Allied health services	63.8 (155)	42.2 (52,164)
Surgical operations	25.1 (61)	22.9 (28,312)
Psychiatrist attendance	2.5 (6)	3.4 (4,153)
Pathology services		
Patient episode initiations	93.0 (226)	88.9 (109,896)
Chemical	84.0 (204)	78.0 (96,374)
Microbiology	80.3 (195)	65.0 (80,366)
Haematology	68.7 (167)	57.9 (71,556)
Tissue	13.6 (33)	12.3 (15,249)
Immunology	9.9 (27)	6.7 (8,285)
Cytopathology	2.5 (6)	1.7 (2,146)
Genetics	1.2 (3)	0.5 (631)
Simple basic tests	0.8 (2)	0.5 (622)
Specimen referred	1.2 (3)	1.7 (2,067)
**Hospitalisation in the 12 months prior to enrolment**		
Emergency department presentations per resident, median (IQR)	1 (1–1)	1 (1–3)
At least 1 emergency department presentation, % (n)	26.2 (73)	44.7 (56,016)
Hospital separations per resident, median (IQR)	1 (1–2)	2 (1–3)
At least 1 hospital separation, % (n)	31.5 (88)	48.2 (60,409)
**Medications prescribed in the 12 months prior to enrolment^**		
Medicines supplied per person, median (IQR)	5 (2,6)	13 (9,18)
At least 1 medication dispensed, % (n)	97.8 (223)	99.3 (141,893)
At least 1 dispensed, % (n)		
Antibiotics	61.0 (139)	74.5 (106,427)
Cefalexin	28.1 (64)	NA
Amoxicillin and clavulanic acid	21.9 (50)	NA
Trimethoprim	19.3 (44)	NA
Amoxicillin	15.8 (36)	NA
Doxycycline	13.2 (30)	NA
Flucloxacillin	4.8 (11)	NA
Clindamycin	4.4 (10)	NA
Nitrofurantoin	4.4 (10)	NA
Ciprofloxacin	3.5 (8)	NA
Methenamine Hippurate	3.1 (7)	NA
Roxithromycin	3.1 (7)	NA
Trimethoprim and sulfamethoxazole	3.1 (7)	NA
Cefaclor	2.6 (6)	NA
Norfloxacin	2.2 (5)	NA
Azithromycin	2.2 (5)	NA
Erythromycin	2.2 (5)	NA
Metronidazole	2.2 (5)	NA
Antivirals	1.8 (4)	1.8 (2,524)
Antimycotics	0.4 (1)	0.5 (686)
Medicines for constipation	36.0 (82)	45.1 (64,434)
Medicines for acid-related disorders	49.1 (112)	51.5 (73,550)
Insulin	7.5 (17)	6.6 (9,393)
Antidiabetics	11.4 (26)	14.2 (20,262)
Opioids	44.7 (102)	48.2 (68,819)
Anti-inflammatory/antirheumatic	7.5 (17)	9.5 (13,649)
Corticosteroids	14.9 (34)	16.2 (23,114)
Other immunosuppressants	0.9 (2)	0.6 (911)
Sex hormones	4.8 (11)	3.5 (4,973)
Lipid-modifying agents	25.4 (58)	36.7 (52,409)
Beta-blocking agents	26.3 (60	28.7 (40,969)
Antidementia	8.3 (19)	10.3 (14,754)
Antidepressants	43.4 (99)	48.3 (68,982)
Psycholeptics	31.1 (71)	47.2 (67,465)

* extracted from aged care funding instrument data

^ missing data GRACE: activities of daily living care requirement, 1.1%; cognition and behaviour care requirement, 2.5%; complex healthcare care requirement, 1.1%; healthcare services, 12.9%; medications, 18.3%

† missing data ROSA: facility location, 0.2%; organisation type, 0.1%; dementia diagnosis, 0.7%; activities of daily living care requirement, 0.7%; cognition and behaviour care requirement, 0.7%; complex healthcare care requirement, 0.7%

NA = data not available

### Care requirements

Care requirements represented by the three ACFI domains were assessed for both datasets (Table 2). Activities of Daily Living (ADL) care requirements were greater in the GRACE cohort compared to ROSA, with 65.9% (n = 184) having a high care requirement for this domain, compared to 54.3% in ROSA (n = 77,552). Cognition and Behaviour care requirements for GRACE were less than those in ROSA, with 47.0% (n = 131) and 60.3% (n = 86,117) having a high care score, respectively. GRACE had a higher proportion of participants with a high care requirement for Complex Healthcare (64.5%, n = 180) compared to the ROSA cohort (53.3%, n = 76,228).

### Utilisation of healthcare services

In the 12 months prior to enrolment/data collection, the proportions of the GRACE and ROSA cohorts that had accessed a MBS-subsidised healthcare service were similar (GRACE = 100%, n = 243; ROSA = 99.4%, n = 122,875). GRACE participants utilised GP services for non-urgent out of hours care most commonly (GRACE = 85.5%, n = 207; ROSA = 54.7%, n = 67,643; Table 2). More GRACE participants accessed urgent out of hours GP services (61.7%, n = 150) compared to ROSA (33.1%, n = 40,904). Both cohorts accessed standard GP attendances similarly (GRACE = 38.7%, n = 94; ROSA = 45.7%, n = 56,465). GRACE participants had team care plans (in which multidisciplinary teams manage a case; GRACE = 81.1%, n = 197; ROSA = 56.0%, n = 69,213) and collaborative domiciliary and residential management reviews (in which a GP and pharmacist review ongoing medication for a resident; GRACE = 61.7%, n = 150; ROSA = 33.7%, n = 41,696) more commonly than those in the ROSA.

GRACE and ROSA cohorts had a similar level of pathology service utilisation, with patient episode initiations the most frequently accessed service for each (GRACE = 93.0%, n = 226; ROSA = 88.9%, n = 109,896; Table 2). Of all pathology services captured, access of microbiology services differed most between the datasets (GRACE = 80.3%, n = 195; ROSA = 65.0%, n = 80,366).

### Hospitalisations

GRACE had a smaller proportion of participants with at least 1 hospitalisation recorded in the 12 months prior to enrolment/data collection (31.5%, n = 88) compared to ROSA (48.2%, n = 60,409). The median number of hospitalisations per resident was similar (GRACE: med = 1, IQR = 1–2; ROSA: med = 2, IQR = 1–3; Table 2). GRACE also had a smaller proportion of participants with at least 1 emergency department presentation in the 12 months prior to enrolment/data collection (GRACE = 26.2%, n = 73; ROSA = 44.7%, n = 56,016).

### Medications

At least 1 medication had been dispensed in the 12 months prior to enrolment/data collection for 97.8% (n = 223) and 99.3% (n = 141,893) of the GRACE and ROSA cohorts, respectively. GRACE participants were taking less medications per person (med = 5, IQR = 2–6) compared to ROSA (med = 13, IQR = 9–18; Table 2). Antibiotics were the most commonly supplied drug class to both cohorts during the 12 months prior to enrolment/data collection, but GRACE had a fewer number of participants who were supplied antibiotics (61.0%, n = 139) compared to ROSA (74.5%, n = 106,427). Psycholeptics were supplied to fewer participants in GRACE (31.1%, n = 71) compared to ROSA (47.2%, n = 67,465).

### Health conditions

The median number of RxRisk conditions per participant did not differ between the two cohorts (both: med = 5, IQR = 3–7; Table 3). In GRACE, the most common RxRisk conditions included gastro-oesophageal reflux disease (49.1%, n = 112), pain (44.7%, n = 102) and depression (43.0%, n = 98). Gastro-oesophageal reflux disease was also the most common condition in ROSA (49.0%, n = 69,977), followed by depression (45.7%, n = 65,351) and hypertension (43.1%, n = 61,542). Compared to ROSA, the GRACE cohort had a higher proportion of participants being treated for osteoporosis/Paget’s disease (GRACE = 23.7%, n = 54; ROSA = 15.6%, n = 22,306) and hypothyroidism (GRACE = 17.5%, n = 40; ROSA = 10.8%, n = 15,450). Most conditions were similar in their prevalence between the two datasets.

**Table 3:** RxRisk-V health conditions for GRACE participants compared to ROSA.

	GRACE (n = 228)	ROSA (n = 142,923)
**RxRisk-V condition, % (n)^**		
Gastro-oesophageal reflux disease	49.1 (112)	49.0 (69,977)
Hyperlipidaemia	25.4 (58)	32.1 (45,927)
Hypertension	40.4 (92)	43.1 (61,542)
Ischaemic heart disease: hypertension	32.9 (75)	35.6 (50,863)
Antiplatelets	19.3 (44)	19.1 (27,258)
Depression	43.0 (98)	45.7 (65,351)
Pain	44.7 (102)	41.2 (58,921)
Anticoagulants	21.9 (50)	17.4 (24,838)
Chronic airways disease	24.1 (55)	22.0 (31,493)
Congestive heart failure	18.4 (42)	17.0 (24,362)
Osteoporosis/Paget	23.7 (54)	15.6 (22,306)
Psychotic illness	13.2 (30)	15.9 (22,680)
Diabetes	15.0 (34)	15.4 (22,025)
Steroid responsive disease	14.0 (32)	10.2 (14,525)
Arrythmia	10.5 (24)	10.1 (14,389)
Anxiety	17.1 (39)	14.5 (20,742)
Glaucoma	11.0 (25)	11.3 (16,120)
Ischaemic heart disease: angina	11.0 (25)	9.7 (13,812)
Dementia	8.3 (19)	16.5 (23,520)
Hypothyroidism	17.5 (40)	10.8 (15,450)
Inflammation/pain	7.5 (17)	5.8 (8,247)
Gout	7.0 (16)	5.9 (8,403)
Parkinson’s disease	11.8 (27)	7.3 (10,435)
Epilepsy	7.0 (16)	9.2 (13,167)
Liver failure	0 (0)	0.1 (81)
Incontinence	3.5 (8)	2.8 (4,063)
Benign prostatic hyperplasia	3.1 (7)	2.8 (4,064)
Malignancies	1.3 (3)	1.5 (2,174)
Renal disease	2.6 (6)	1.1 (1,630)
Hyperthyroidism	0 (0)	0.9 (1,304)
Allergies	0 (0)	0.6 (842)
Migraine	0 (0)	0.6 (810)
Irritable bowel syndrome	0.4 (1)	0.6 (831)
Smoking cessation	0.4 (1)	0.4 (581)
Pancreatic insufficiency	0 (0)	0.4 (547)
Psoriasis	1.3 (3)	0.5 (655)
Bipolar disorder	0 (0)	0.5 (675)
Transplant	0 (0)	0.1 (145)
Alcohol dependency	0 (0)	< 0.1 (39)
Pulmonary hypertension	0 (0)	< 0.1 (37)
Hepatitis B	0 (0)	< 0.1 (19)
HIV	0 (0)	< 0.1 (38)
Hyperkalaemia	0 (0)	< 0.1 (20)
Malnutrition	0 (0)	0 (0)
Tuberculosis	0 (0)	< 0.1 (< 5)
Hepatitis C	0 (0)	< 0.1 (10)
Number of conditions per person, median (IQR)	5 (3,7)	5 (3,7)

^ missing data GRACE, 18.3%

### Additional GRACE datapoints

Some characteristics could not be compared between the GRACE and ROSA cohorts and these are summarised in **Supplementary Table 3**. Most GRACE participants were staying in their own rooms (97.8%; n = 273), with a small proportion also living in memory support areas (12.9%; n = 36). Diet type and texture was highly conserved among participants, with 93.9% (n = 262) reporting a normal diet, 72.8% reporting normal meal texture (n = 203) and 91.4% (n = 255) reporting a normal liquid texture. Most participants were receiving a standard fortified diet (56.3%; n = 157), but a large proportion were receiving a high energy high protein supplemented diet (39.4%; n = 110). Seven (2.5%) participants reported a colostomy/ileostomy. No GRACE participants reported a urinary catheter in situ, vascular catheter in situ, urostomy, or a tracheostomy hence they are not included in **Supplementary Table 3**. Seventy-two (26.0%) participants were receiving wound care at the time of enrolment, with grade 1–2 pressure ulcers the most common wound being treated (6.5%; n = 18). Using the pre-calculated PAS-CIS method, GRACE participants were most commonly assigned a moderate impairment score (39.8%; n = 111), followed by severe (28.0%; n = 78) and mild impairment (27.6%, n = 77). Very few were assigned no to minimal impairment (2.9%; n = 8).

## Discussion

The primary strength of the GRACE study is the combination of comprehensive demographic, health care, health status, medical, pharmaceutical, and facility variables with intestinal and oropharyngeal microbiome and resistome data for permanent residents of RACFs. Together, we have designed and conducted the first large-scale metagenomic assessment of gut microbiome and resistome characteristics in residents of long-term aged care. The research design developed to establish this cohort has enabled powerful opportunities for novel and extensive investigations currently underway into relationships between risk factors in aged care, current practices in aged care, intestinal health, and disease state outcomes, for a critically understudied and vulnerable population. For example, future work will include investigations assessing associations between cognitive and behavioural diagnoses and the composition of the microbiota to measure the impact of variables in aged care on the increasing burden of cognitive decline.

Another key strength from GRACE was the high rate of recruitment (75%) from residents and families of residents in RACFs. This was most likely attributable to the availability of a research nurse in the study team who personally and extensively communicated with residents and their families. For enhanced rates of recruitment, future studies may also benefit from dedicating significant resources towards communication strategies, particularly when involving elderly populations.

A limitation of the GRACE study resulted from the impact of the SARS-CoV-2 (COVID-19) pandemic. However, whilst this affected the final sample size, the severity of the effect was dampened by the high recruitment rate of participants. In addition, the location of participating RACFs should be considered. These were exclusively in metropolitan areas and may therefore differ in their characteristics from those located in rural or more remote areas, in particular access to healthcare services and resident demographics. Similarly, only not-for-profit aged care providers participated in the study, who may have had higher staffing capabilities and different approaches to food provisions compared to other types of facilities. Subsequent studies would benefit from a diversified cross-section of RACFs in both geographical location and funding type. Data relating to resident ethnicity was also not captured.

The potential contribution of participant selection biases must also be considered. Residents who were in respite care or receiving palliative/end-of-life care were excluded, given the considerable burden already experienced by these individuals, and inability to access follow-up data. Whilst unavoidable, this potentially influenced the degree to which the cohort is representative of an entire facility. Differences in individuals who consented to participation, compared to those who did not, might also represent a source of bias, although no specific differential characteristics were evident. Lastly, data provided by participating facilities might also be incomplete or contain inaccuracies.

Specifically in relation to the emergence of antibiotic resistance, potential risk exposures, including high antibiotic use, high medication usage, a high proportion of health conditions experienced, and frequent access of after-hours GP services, were identified in the GRACE cohort, and were reflective of exposures in the wider residential aged care population.

In conclusion, we have assembled a cohort of aged care residents that is broadly representative of the Australian aged care population to provide a platform for future analysis. Specifically, metagenomic data isolated from participant clinical samples and samples from the built environment will be used to investigate microbiome and resistome characteristics at both resident and facility levels. Individual and facility risk exposures will be aligned with metagenomic data to identify principal determinants for antimicrobial resistance carriage. Ultimately, these analyses will inform measures aimed at reducing the emergence and spread of antimicrobial resistant pathogens in this high-risk population.

### Collaboration

The GRACE team have established a cohort with comprehensively detailed information on overall health, microbiome profiles, and medication use in RACFs. The primary aim for establishing this cohort was to investigate the existence and spread of resistant bacteria in residential aged care to help improve facility management, prevent the spread of harmful bacteria, and ultimately improve the health of aged care residents and the wider community. The authors welcome approaches from other researchers to discuss the potential for collaborative studies that utilise this valuable resource.

### Electronic supplementary material

Below is the link to the electronic supplementary material.

Supplementary Material 1

## Data Availability

The GRACE study data are available upon reasonable request. GRACE study data described in this article are available to all interested researchers through collaboration. Please contact GBR (geraint.rogers@sahmri.com). Metagenomic sequencing data will be made available via a public registry once completed. Due to data custodian restrictions related to the sharing of linked data in ROSA, these data cannot be made publicly available to other researchers.

## Declarations

### Patient and public involvement

RACF staff provided advice on how to engage with participants, families, and other staff members, and the formulation of the participant information data capture instrument. Although residents were not involved in the design, recruitment, conduct, reporting, or dissemination of this study directly, resident committees were involved through presentations at their respective sites. Ongoing feedback is provided to aged care providers and their participating residents via printed, electronic, and in-person communication.

### Provenance and peer review

Not commissioned.

## Abbreviations

ACFI: Aged Care Funding Instrument
AMR: Antimicrobial Resistance
DHS: Department of Human Services
GP: General Practitioner
GRACE: Generating evidence on antimicrobial Resistance in the Aged Care Environment
MBS: Medicare Benefits Schedule
MDRO: Multi-Drug Resistant Organism
PAS-CIS: Psychogeriatric Assessment Scales – Cognitive Impairment Scale
PBS: Pharmaceutical Benefits Scheme
RACF: Residential Aged Care Facility
ROSA: Registry of Senior Australians
